# Hydrogen Sulfide as a Novel Regulatory Factor in Liver Health and Disease

**DOI:** 10.1155/2019/3831713

**Published:** 2019-01-20

**Authors:** Dong-Dong Wu, Da-Yong Wang, Hui-Min Li, Jian-Cheng Guo, Shao-Feng Duan, Xin-Ying Ji

**Affiliations:** ^1^School of Basic Medical Sciences, Henan University, Kaifeng, Henan 475004, China; ^2^Henan International Joint Laboratory for Nuclear Protein Regulation, Henan University, Kaifeng, Henan 475004, China; ^3^The First Affiliated Hospital of Henan University, Kaifeng, Henan 475001, China; ^4^Center for Precision Medicine, Zhengzhou University, Zhengzhou, Henan 450052, China; ^5^Institute for Innovative Drug Design and Evaluation, School of Pharmacy, Henan University, Kaifeng, Henan 475004, China

## Abstract

Hydrogen sulfide (H_2_S), a colorless gas smelling of rotten egg, has long been recognized as a toxic gas and environment pollutant. However, increasing evidence suggests that H_2_S acts as a novel gasotransmitter and plays important roles in a variety of physiological and pathological processes in mammals. H_2_S is involved in many hepatic functions, including the regulation of oxidative stress, glucose and lipid metabolism, vasculature, mitochondrial function, differentiation, and circadian rhythm. In addition, H_2_S contributes to the pathogenesis and treatment of a number of liver diseases, such as hepatic fibrosis, liver cirrhosis, liver cancer, hepatic ischemia/reperfusion injury, nonalcoholic fatty liver disease/nonalcoholic steatohepatitis, hepatotoxicity, and acute liver failure. In this review, the biosynthesis and metabolism of H_2_S in the liver are summarized and the role and mechanism of H_2_S in liver health and disease are further discussed.

## 1. Introduction

Hydrogen sulfide (H_2_S) is a colorless and water-soluble gas with the characteristic foul odor of rotten egg [1–3]. At physiological pH, nearly two thirds of H_2_S exists as H^+^ and hydrosulfide anion, which subsequently decomposes to H^+^ and sulfide ion [4]. In mammals, H_2_S is produced from L-cysteine and L-homocysteine mainly by cystathionine *γ*-lyase (CSE) and cystathionine *β*-synthase (CBS). Both CSE and CBS are cytosolic enzymes [5, 6]. 3-Mercaptopyruvate sulfurtransferase (3-MST) acts in combination with cysteine aminotransferase (CAT) to produce H_2_S from L-cysteine in the presence of *α*-ketoglutarate (*α*KG). 3-MST and CAT are located in the mitochondria and cytosol [3, 7]. Furthermore, a recent study has shown that D-amino acid oxidase could metabolize D-cysteine to an achiral *α*-ketoacid, 3-mercaptopyruvate (3-MP), which is further metabolized to H_2_S by 3-MST in both kidney and brain [8].

**Figure 1:**
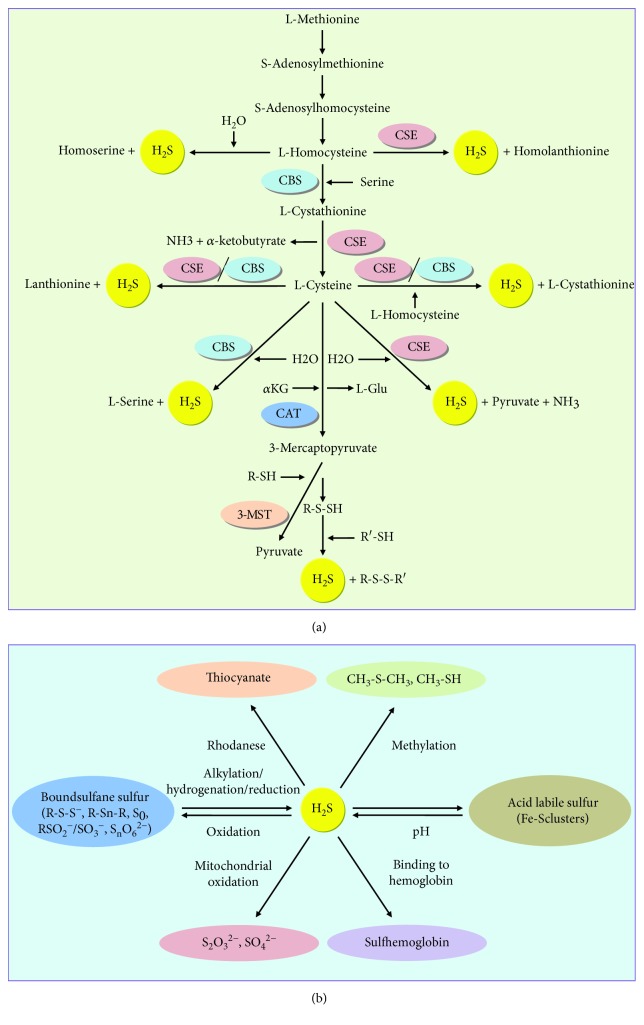
A schematic illustration of the biosynthesis and metabolism of H_2_S in the liver. (a) H_2_S is enzymatically produced from L-cysteine and L-homocysteine by CSE and CBS. 3-MST acts in combination with CAT to produce H_2_S from L-cysteine in the presence of *α*KG. (b) H_2_S can be stored as acid-labile sulfur and bound sulfane sulfur. Catabolism of H_2_S is thought to occur mainly via rhodanese, methylation, binding to hemoglobin, and mitochondrial oxidation. H_2_S: hydrogen sulfide; CSE: cystathionine *γ*-lyase; CBS: cystathionine *β*-synthase; H_2_O: water; NH_3_: ammonia; *α*KG: *α*-ketoglutarate; L-Glu: L-glutamate; CAT: cysteine aminotransferase; 3-MST: 3-mercaptopyruvate sulfurtransferase; pH: potential of hydrogen; CH_3_-S-CH_3_: dimethyl sulfide; CH_3_-SH: methanethiol; S_2_O_3_^2−^: thiosulfate; SO_4_^2−^: sulfate.

H_2_S has been considered the third gaseous signaling molecule that plays important regulatory roles in a number of physiologic conditions, such as angiogenesis [9], vasodilatation [10], and neuronal activity [11]. The liver, the largest solid organ in the body, plays a key role in glucose and lipid metabolism, antioxidant defense, and xenobiotic metabolism [12–14]. The liver is an important organ for H_2_S production and its clearance [3, 15]. CSE, CBS, and 3-MST have been detected in the liver, and they contribute to liver production of H_2_S to different extents [3, 12]. The production and catabolism of H_2_S in the liver are shown in Figure 1. Hepatic H_2_S is involved in mitochondrial biogenesis and bioenergetics, insulin sensitivity, lipoprotein synthesis, and glucose metabolism [12, 16, 17]. However, H_2_S also contributes to the pathogenesis and treatment of many liver diseases, such as liver cirrhosis [18], liver cancer [19], hepatic fibrosis [20], hepatic ischemia/reperfusion (I/R) injury [21], and nonalcoholic steatohepatitis (NASH) [22].

In the present review, we highlight recent studies that provide new insight into the biosynthesis and metabolism of H_2_S in the liver and further discuss the role and mechanism of H_2_S in liver health and disease.

## 2. H_2_S in Hepatic Function

### 2.1. H_2_S in Hepatic Oxidative Stress

Reactive oxygen species (ROS), the by-products of normal aerobic cellular metabolism, are considered to be important signaling molecules in many cellular processes, including cell adhesion, immune response, apoptosis, and cell survival and growth [23–25]. Oxidative stress means that an imbalance develops between ROS and antioxidant systems, which is implicated in liver cancer [26], fatty liver [22], liver failure [27], and hepatic ischemia/reperfusion [28]. It has been demonstrated that increased carbonyl formation is an indicator of oxidative stress [12]. The level of carbonyl formation in the liver of CBS-deficient mice is higher when compared to the control group [29], suggesting that CBS may play a role in reducing hepatic oxidative stress. Recent studies have shown that treatment with relatively low concentrations of H_2_S donor (NaHS or Na_2_S) could decrease ROS levels, lipid peroxidation, and cytochrome P450 2E1 (CYP2E1) activity and elevate glutathione (GSH) levels and antioxidative enzyme activities like superoxide dismutase, glutathione peroxidase, catalase, and glutathione S-transferase in hepatocytes [30–32]. It should be noted that administration of 500 *μ*M NaHS could increase ROS formation through the inhibition of cytochrome c oxidase and the depletion of GSH in rat primary hepatocytes, which could lead to hepatotoxicity [33]. These results together indicate that relatively low levels of H_2_S could protect against hepatic oxidative stress; however, relatively high concentrations of H_2_S may exert opposite effects. A proper dose of H_2_S should be adopted to avoid H_2_S-induced cytotoxicity in normal liver cells when it is used for the treatment of liver diseases.

### 2.2. H_2_S in Hepatic Glucose Metabolism

The liver is crucial for the maintenance of blood glucose homeostasis by uptake of glucose in the postprandial state and conversion to triglyceride and glycogen and by production of glucose in the postabsorptive state by gluconeogenesis and glycogenolysis [34, 35]. Defects in the mechanisms by which insulin and glucose regulate hepatic glycogen metabolism disrupt blood glucose homeostasis and lead to metabolic disorders such as diabetes [35, 36] and glycogen storage disease [37]. It has been shown that the CSE activity is lower in livers of type 1 diabetic rats and peripheral blood mononuclear cells of type 1 diabetic patients [38], indicating that H_2_S is involved in glucose regulation [17, 39]. A recent study demonstrates that the rate of gluconeogenesis in CSE knockout mice is reduced, which can be reversed by administration of NaHS [40]. Similarly, incubation with NaHS impairs glucose uptake and glycogen storage via decreasing glucokinase activity and increasing gluconeogenesis through S-sulfhydration of pyruvate carboxylase in hepatocytes [16, 41]. These findings suggest that H_2_S may be a potential target in the treatment of diabetes.

### 2.3. H_2_S in Hepatic Lipid Metabolism

The liver is the main metabolic organ and plays an important role in fatty acid and cholesterol metabolism [42]. Hepatic lipid metabolism is orchestrated by a delicate interplay of hormones, transcription factors, nuclear receptors, and intracellular signaling pathways [43]. Excessive accumulation of fat in the liver disturbs its function and leads to the development of many liver diseases, such as NASH, liver cirrhosis, and liver cancer [44]. CBS deficiency in mice liver increases expression of genes induced by endoplasmic reticulum stress and genes that regulate the expression of enzymes required for cholesterol and fatty acid biosynthesis and uptake [45]. Another study indicates that the levels of triglyceride and nonesterified fatty acid are elevated and the activity of thiolase, a key enzyme in beta-oxidation of fatty acids, is decreased in the liver of CBS-deficiency mice [46]. It has been shown that the expression levels of CBS and CSE and the lipid peroxidation were increased in the liver of high-fat diet- (HFD-) fed mice [47]. In addition, tyrosol supplementation increases hepatic CSE and CBS expression and H_2_S synthesis in HFD-fed mice, which is associated with the attenuation of HFD-induced hepatic lipid peroxidation [48]. A recent study has revealed that administration of NaHS decreases the accumulation of lipids such as total cholesterol and triglyceride through downregulation of fatty acid synthase and upregulation of carnitine palmitoyltransferase-1 in the liver of HFD-induced obese mice [49]. S-Propargyl-cysteine (SPRC), a substrate for endogenous H_2_S, could reduce the lipid content both in human hepatocellular carcinoma HepG2 cells and in the liver of mice with nonalcoholic fatty liver disease (NAFLD) [50]. These findings indicate that H_2_S is involved in hepatic lipid metabolism and the underlying mechanisms are needed to be further investigated.

### 2.4. H_2_S in Hepatic Vasculature

The liver has a complex system of vascular supply, including the inflow of oxygenated blood through the hepatic artery and deoxygenated blood through the portal vein, as well as the outflow of deoxygenated blood through the hepatic veins to the inferior vena cava [51]. Anatomical variations in hepatic artery are of importance to surgeons in planning effective therapeutic strategies for abdominal surgical procedures [52]. The hepatic artery is involved in the pathogenesis of several diseases, such as stenosis, thrombosis, aneurysm, and pseudoaneurysm [51]. H_2_S plays a key role in vascular homeostasis during physiological and pathological conditions. H_2_S-based therapy in vascular disease is a novel area of research [53]. H_2_S acts as an autocrine mediator in regulation of the contraction of hepatic stellate cells (HSCs) and that a decreased expression of CSE in HSCs may lead to the increased intrahepatic resistance in rodent models of liver cirrhosis [18]. A recent study has shown that H_2_S differentially contributes to the microcirculatory dysfunction in both systemic and hepatic microcirculations, which can be attributed to H_2_S-induced differential vasoactive function on sinusoidal and presinusoidal sites within the liver [54]. Another study demonstrates that H_2_S increases the hepatic arterial buffer capacity and mediates vasorelaxation of the hepatic artery through activation of K_ATP_ channels [55]. However, a vasoconstrictor action of H_2_S on the hepatic sinusoid has been observed, which is different from the dilatory effect of H_2_S in presinusoidal resistance vessels [56]. More efforts should be paid to validate the different effects of H_2_S on hepatic vasculature.

### 2.5. H_2_S in Hepatic Mitochondrial Function

Mitochondria are double-membrane organelles whose shape is instrumental to their function in many cellular processes [57]. The major role of mitochondria is to regulate the production of energy-rich molecules such as adenosine triphosphate [58]. Mitochondria play important roles in the metabolism of glucose, lipids, and protein in the liver [59]. Under normoxic conditions, the protein expression of CBS in liver mitochondria is at a low level. Hepatic ischemia/hypoxia results in the accumulation of CBS in mitochondria and increased H_2_S production, which prevents hypoxia-induced mitochondrial ROS production and Ca^2+^-mediated cytochrome C release from mitochondria [60]. CSE-generated H_2_S induces liver mitochondrial biogenesis, which can be attributed to the peroxisome proliferator-activated receptor-*γ* coactivator-1*α* and peroxisome proliferator-activated receptor-*γ* coactivator-related protein signaling in primary hepatocytes [61]. 3-MP, the substrate of the enzyme 3-MST, stimulates mitochondrial H_2_S production and enhances hepatic mitochondrial electron transport and cellular bioenergetics at low concentration, while it inhibits cellular bioenergetics at a higher concentration. In addition, low concentration of H_2_S induces a significant increase in hepatic mitochondrial function, while a higher concentration of H_2_S is inhibitory [62]. These results indicate that endogenous H_2_S plays a physiological role in the maintenance of mitochondrial electron transport and cellular bioenergetics. Considering that different concentrations of exogenous H_2_S exert diverse effects on hepatic mitochondrial function, the proper dose range of exogenous H_2_S should be confirmed to achieve optimal hepatic mitochondrial function.

### 2.6. H_2_S in Hepatic Differentiation

A number of etiologies such as viral infections, toxic injury, and genetic or autoimmune disorders may cause severe liver dysfunction resulting in acute liver failure or chronic liver disease [63]. Liver transplantation is the primary method to treat acute liver failure and end-stage liver diseases. However, it is limited by numerous problems, including shortage of donor organs, high cost, and immune rejection [64]. To solve these problems, stem-cell-based therapeutic strategies have emerged as alternative options [63, 65]. A recent study indicates that physiological concentrations of H_2_S could increase the ability of human tooth-pulp stem cells (HTPC) to undergo hepatogenic differentiation [66]. Another study has revealed that H_2_S increases hepatic differentiation of both HTPC and human bone marrow stem cells [67]. These cells may be suitable for generation of functionally useful hepatocytes and transplantation into model animals with liver diseases. Whether H_2_S can play a role in hepatic differentiation of other types of cells needs to be further investigated.

### 2.7. H_2_S in Hepatic Circadian Rhythm

The circadian clock system comprises peripheral clocks in peripheral tissues and a central clock located in the suprachiasmatic nucleus of the hypothalamus [68]. Peripheral clocks in the liver contribute to maintaining liver homeostasis, including the regulation of energy metabolism and the expression of enzymes controlling the absorption and metabolism of xenobiotics [69]. Clock dysfunction leads to the development of liver diseases such as fatty liver diseases, hepatitis, cirrhosis, and liver cancer, and these disorders also disrupt clock function [68, 70]. A recent study has shown that treatment with NaHS could maintain the circadian rhythm of clock gene in isolated liver cells. It is speculated that H_2_S increases the activity of sirtuin 1 protein and changes the nicotinamide adenine dinucleotide+/reduced form of nicotinamide adenine dinucleotide ratio in hepatocytes to maintain the rhythm of expression of circadian clock genes, which can prevent and treat lipid metabolism-related diseases caused by the biological clock disorders [71]. In light of the key role of H_2_S in regulating hepatic circadian rhythm, further studies are needed to elucidate whether H_2_S could relieve liver diseases through hepatic circadian rhythm.

### 2.8. Natural Sulfur-Containing Agents in Hepatic Function

Garlic (*Allium sativum*), a member of the lily family, has been widely used both as a foodstuff and a traditional medicine worldwide for many centuries [72–74]. Garlic oil, one of the garlic products, is usually prepared by steam distillation and has been shown to contain a number of organosulfur compounds, such as diallyl sulfide (DAS), diallyl disulfide (DADS), and diallyl trisulfide (DATS), which have been considered to be the major biological agents [75, 76]. It has been reported that DAS activates nuclear receptor CAR to induce the *Sult1e1* gene in the mouse liver. Whether DAS can play a role in estradiol synthesis pathways, estradiol turnover, or expression/activity of SULT1E1 in other tissues/organs needs to be clarified [74]. Another study indicates that administration of DADS or DATS increases the activities of the phase II enzymes, quinone reductase and glutathione S-transferase, and antioxidative enzyme glutathione peroxidase in rat liver cytosol, suggesting that DADS/DATS could increase the detoxification and antioxidant effects of the liver [77]. Similarly, DADS and DATS have been shown to increase the activities of both GSH reductase and GSH S-transferase in rat livers [78]. Furthermore, a recent study has shown that aldehyde dehydrogenase activity can be inhibited *in vivo* in the rat liver after treatment with DATS [79]. These results together indicate that natural sulfur-containing agents may play important roles in the regulation of hepatic function. Recent studies have demonstrated that DATS, DADS, and DAS can act as H_2_S donors [80, 81]. Whether the regulatory effects of DATS, DADS, and DAS are mediated by H_2_S need to be further investigated.

## 3. H_2_S in Hepatic Injury

### 3.1. H_2_S in Hepatic Fibrosis

Hepatic fibrosis results from chronic damage to the liver in conjunction with the excessive accumulation of the extracellular matrix (ECM) of predominantly type I collagen [82]. A variety of factors such as viral infections, alcohol abuse, genetic abnormalities, overload of metal ions, and autoimmunity contribute to hepatic fibrosis [82, 83]. Hepatic fibrosis is the inevitable pathological process of many chronic liver diseases, including NASH, NAFLD, and viral hepatitis [84]. Once these chronic diseases aggravate further, hepatic fibrosis may progress to liver cirrhosis or hepatocellular carcinoma (HCC) [85]. There is increasing evidence that activated hepatic stellate cells (HSCs) are the central effector cells, which play key roles in the excessive synthesis and deposition of ECM in hepatic interstitium, leading to hepatic fibrosis [82]. Despite the development made in this field, there are limited available treatments for this disease [86, 87]. It is urgent to develop novel therapeutic drugs aimed at attenuating or preventing hepatic fibrosis. It has been reported that CBS deficiency promotes fibrosis, oxidative stress, and steatosis in mice liver, suggesting that H_2_S is involved in hepatic fibrosis [29]. Furthermore, recent studies have shown that H_2_S could attenuate hepatic fibrosis both *in vivo* and *in vitro* (Table 1). Therefore, H_2_S may be a promising therapeutic target for the treatment of a variety of fibrotic diseases. The expression levels and roles of H_2_S-generating enzymes in fibrotic diseases need to be further determined. Furthermore, proper H_2_S-releasing agents can be designed and developed to treat fibrotic diseases in a controlled way.

### 3.2. H_2_S in Liver Cirrhosis

Liver cirrhosis is an increasing cause of morbidity and mortality, particularly in developed countries [92]. Liver cirrhosis is a serious condition in which scar tissue replaces the healthy tissue of the liver and regenerative nodules surrounded by fibrous bands in response to the injury [93]. Cirrhosis is the common end of progressive liver disease of various causes, leading to several chronic liver failure entailing complications including peritonitis, hepatic encephalopathy, spontaneous bacterial ascites, and esophageal varices [94]. The major clinical consequences of cirrhosis are impaired liver function, an increased intrahepatic resistance, and the development of HCC [93, 95]. In spite of current advancements in the treatment, orthotopic liver transplantation remains the only definite solution to end-stage cirrhosis [92, 94, 96]. Several studies have demonstrated that the mRNA and protein levels of hepatic CSE and the serum levels of H_2_S in rats are decreased in the cirrhosis group compared with those in the control group [18, 97, 98]. A hypothesis suggests that H_2_S may contribute to the pathogenesis of vascular dysfunction in cirrhosis [99]. In addition, treatment with NaHS could attenuate CCl_4_-induced liver cirrhosis, hepatotoxicity, and portal hypertension through anti-inflammation, antifibrosis, and antioxidation effects in rats, suggesting that targeting H_2_S may present a promising approach in alleviating liver cirrhosis and portal hypertension [31]. However, more studies are urgently needed to clarify the role and mechanism of H_2_S in different animal models of liver hepatitis.

### 3.3. H_2_S in Liver Cancer

Malignant liver tumors can be classified as primary or secondary (metastatic) [100]. Primary malignancies of the liver are HCC, which is the sixth most common cancer and the third leading cause of cancer-related death worldwide [101, 102]. The main etiologic factors for HCC are chronic hepatitis B virus and hepatitis C virus infection, NAFLD, and alcoholic cirrhosis [103]. Most patients with HCC are diagnosed at a late stage when curative treatments are not applicable, and the majority of death is due to tumor recurrence [104]. Thus, it is urgent to uncover novel etiological mechanisms and develop more effective approaches for the prevention and treatment of HCC [105]. In the liver, biosynthesis and clearance of H_2_S mainly occur in hepatic stellate cells, the major cell source of the extracellular matrix in liver fibrosis and HCC [106]. It has been shown that CSE is overexpressed in human hepatocellular carcinoma HepG2 and PLC/PRF/5 cells and contributes to the proliferation of human HCC cells [107]. Similarly, another study indicates that CSE/H_2_S promotes human HCC cell proliferation via cell cycle progression regulation [19]. Furthermore, CBS is overexpressed in human hepatocellular carcinoma HepG2 and SMMC-7721 cells and inhibition of endogenous CBS/H_2_S could reduce the viability and proliferation of SMMC-7721 cells [108]. Moreover, administration of 500 *μ*mol/L NaHS could induce cell proliferation, migration, and angiogenesis and exhibit antiapoptotic effects in PLC/PRF/5 hepatoma cells via activation of the nuclear factor-*κB* (NF-*κ*B) pathway [109]. However, treatment with 10^−3^ M NaHS inhibits HCC cell migration, proliferation, and division through induction of cell apoptosis [106]. *P-*(4-methoxyphenyl)-*p-*4-morpholinylphosphinodithioic acid morpholine salt (GYY4137)-mediated suppression of cell proliferation in human HCC cells may be due to direct targeting of the signal transducer and activator of the transcription 3 pathway [110]. A recent study has demonstrated that the growth and migration of human HCC cells are enhanced by 10-100 *μ*M NaHS and dose-dependently inhibited by 600-1000 *μ*M NaHS through epidermal growth factor receptor/extracellular signal-regulated protein kinase/matrix metalloproteinase 2 and phosphatase and tensin homolog deleted on chromosome ten/protein kinase B (PKB/AKT) signaling pathways [111]. Taken together, these results indicate that endogenous H_2_S or relatively low levels of exogenous H_2_S may promote the growth of HCC cells, while treatment with higher concentrations of exogenous H_2_S may exhibit anticancer effects. Therefore, knockdown/knockout of H_2_S-generating enzymes in cancer cells and development of H_2_S-releasing donors/drugs may be promising strategies for anticancer therapy.

### 3.4. H_2_S in Hepatic I/R Injury

Hepatic I/R injury is a major complication in many clinical scenarios, such as liver transplantation, trauma, hemorrhagic shock and resuscitation, liver resection, and aortic injury during abdominal surgery [112–114]. Hepatic I/R injury leads to acute or chronic liver failure and increases the rate of morbidity and mortality [115]. Under different pathological conditions, hepatic I/R injury can be classified into warm and cold I/R injury according to the environmental temperature [115]. It is well known that hepatic I/R injury involves several mechanisms, including pH imbalance, calcium overload, mitochondrial dysfunction, ROS overproduction, anaerobic metabolism, activation of Kupffer cells and neutrophils, and the production of cytokines and chemokines [113, 114, 116, 117]. Despite significant improvements in surgical techniques and perioperative care, therapies to suppress hepatic I/R injury at the bedside remain limited largely due to the complex mechanisms [118]. Therefore, there is a clear need for the development of novel agents to protect the liver from I/R injury. An increasing number of studies suggest that H_2_S could attenuate hepatic I/R injury in several ways, such as antioxidation, anti-inflammation, antiapoptosis, and AKT activation (Table 2). These results indicate that H_2_S plays an important role in attenuating hepatic I/R injury, and targeting H_2_S may present a promising approach against I/R-induced liver injury. However, it should be noted that elevated endogenous H_2_S could not alleviate hepatic I/R injury in insulin-resistant rats, whereas silymarin preconditioning is able to prevent oxidative, inflammatory, nitrosative, and apoptotic injuries associated with hepatic I/R, which can be attributed to the suppression of endogenous H_2_S production [129]. Furthermore, a recent study suggests that brief and repeated ischemic postconditioning (IPoC) could increase the expression of CSE after I/R in diabetes mellitus, and the modulation of CSE may contribute to the renoprotective effect of IPoC [130]. Whether the expression levels of H_2_S-generating enzymes in hepatic I/R injury are increased need to be further investigated.

### 3.5. H_2_S in NAFLD/NASH

NAFLD affects approximately 25% of the general adult population and is currently the most common cause of chronic liver disease worldwide [131, 132]. NAFLD is defined as the presence of >5% steatosis, no significant alcohol consumption, and no competing etiologies for hepatic steatosis [133]. Development of NAFLD is associated with metabolic syndrome, such as diabetes, obesity, and dyslipidemia [134]. NASH is considered the progressive form of NAFLD and is characterized by inflammation, hepatocellular injury, liver steatosis, and different degrees of fibrosis [135]. Despite intensive investigations, there are currently no approved therapies for NAFLD/NASH. Therefore, there is an unmet need for developing novel and effective treatments for NAFLD/NASH. Methionine is the most toxic amino acid in mammals. It has been reported that excessive methionine intake induces acute lethal hepatitis in mice lacking CSE [136]. Another study indicates that free fatty acids upregulate hepatic expression of 3-MST and subsequently inhibit the CSE/H_2_S pathway, leading to NAFLD [137]. In addition, exercise training can restore bioavailability of H_2_S and promote autophagy influx in livers of mice fed with HFD. Recently, a growing number of studies have shown that H_2_S could play important roles in NAFLD/NASH (Table 3). Novel H_2_S donors and H_2_S-releasing drugs can be designed and applied for the treatment of NAFLD/NASH.

### 3.6. H_2_S in Hepatotoxicity

Hepatotoxicity refers to liver injury induced by different types of prescription or nonprescription drugs, such as biological agents, natural medicines, health products, dietary supplements, traditional Chinese medicines (TCMs), and small chemical molecules [140]. TCMs are abundant sources of biologically active substances which have been widely used in the prevention and treatment of human diseases [141–143]. However, an increasing number of studies have shown that TCMs could induce severe adverse effects, such as hepatotoxicity [143–145]. Hepatotoxicity is the leading cause of acute liver failure in the clinic and the main reason that drugs are taken off the market [146]. The wide range of culprit agents and lack of objective diagnostic tests lead to many challenges in the diagnosis and management of hepatotoxicity [147]. In spite of its low incidence in the general population, the possibility of hepatotoxicity in patients with unexplained acute/chronic liver injury needs to be considered [147, 148]. A recent study demonstrates that uranium (U) intoxication decreases endogenous H_2_S generation in the hepatic homogenates, while administration of NaHS can reduce U-induced acute hepatotoxicity through antioxidant and antiapoptotic signaling pathways in rats [32]. Acetaminophen overdose is one of the leading causes of drug-induced acute liver failure [149]. H_2_S treatment alleviates acetaminophen hepatotoxicity in mice partly through antioxidative and anti-inflammatory effects [150]. Another study indicates that H_2_S anions could protect against acetaminophen-induced hepatotoxicity by directly scavenging reactive N-acetyl-p-benzoquinone imine [151]. Thus, H_2_S has a potential therapeutic value for the treatment of hepatotoxicity.

### 3.7. H_2_S in Acute Liver Failure (ALF)

ALF is a rare multiorgan-failure disease that is usually caused by viral hepatitis, ingestion of drugs or toxic substances, or hepatic I/R injury [152]. ALF could lead to rapid deterioration of liver function with subsequent coagulopathy and encephalopathy [153]. ALF patients often require and undergo orthotopic liver transplantation or die due to shortage of donor livers [152]. The major problem in the treatment of ALF is the lack of suitable mechanistic biomarkers and broad-spectrum anti-ALF agents [154]. It has been reported that inhibition of CSE or administration of sodium thiosulfate protects against ALF by increasing thiosulfate levels and upregulating antioxidant and antiapoptotic defense in the liver [27]. Similarly, CSE deficiency protects against the development of multiorgan failure and attenuates the inflammatory response in a murine model of burn [155]. These results suggest that CSE may be a potential therapeutic target in ALF. Whether CBS or 3-MST deficiency can exert similar effects needs to be further investigated.

### 3.8. Natural Sulfur-Containing Agents in Hepatic Injury

An increasing number of studies have shown that the garlic constituents possess various biological activities, including anticarcinogenesis, antioxidative, antimicrobial, antihypertensive, antithrombotic, hypolipidemic, radioprotective, immunomodulatory, antidiabetic, and anti-inflammatory effects [75, 156–158]. As can be seen in Table 4, many natural sulfur-containing agents could protect against hepatotoxicity mainly through antioxidative, anti-inflammatory, and antiapoptotic effects. Recent studies have shown that DATS possesses a hepatoprotective effect against carbon tetrachloride- (CCl_4_-) induced liver injury and ethanol-induced hepatic steatosis in rats [166–169]. DADS can activate the HO-1/Nrf2 pathway, which may contribute to the protective effects of DADS against ethanol-induced liver injury [170]. Another study demonstrates that DADS increases the levels of phase II/antioxidant enzymes and decreases the levels of inflammatory mediators in CCl_4_-induced liver injury [158]. Protective effects of DAS were also observed in lipopolysaccharide/D-galactosamine/mercuric chloride-induced hepatic injury in rats [171, 172]. Furthermore, a recent study reveals that DATS can inhibit the profibrogenic properties and alleviate oxidative stress in hepatic stellate cells through the production of H_2_S [91]. Moreover, an increasing number of studies have indicated that DATS, DADS, and DAS could inhibit the growth of human liver cancer cells [173–178]. More efforts should be made to determine the mechanisms of action of natural sulfur-containing agents on liver diseases, such as liver cirrhosis, hepatic I/R injury, and NAFLD/NASH.

## 4. Conclusions

The liver plays a key role in glucose and lipid metabolism, antioxidant defense, and xenobiotic metabolism. The liver is one of the major organs for the production and metabolism of H_2_S. CSE, CBS, and 3-MST are three main H_2_S-generating enzymes, and they contribute to the production of H_2_S to different extents in the liver. Whether the liver could produce H_2_S via another enzyme/pathway needs to be further investigated and confirmed. H_2_S is the third gaseous signaling molecule that is involved in glucose and lipid metabolism, cell differentiation, and circadian rhythm in the liver. Further studies are needed to determine the effects of endogenous H_2_S on hepatic physiological processes. It is worth noting that H_2_S could exhibit two obviously opposite effects on hepatic vasculature, oxidative stress, and mitochondrial function, which can be attributed to the concentration, time frame, and reaction time of H_2_S, as well as the differences between disease stages or models. In light of the important roles of nitric oxide (NO) and carbon monoxide (CO) in mammalian biology, whether H_2_S exerts the regulatory effects by interacting with NO and/or CO should be clarified.

**Figure 2:**
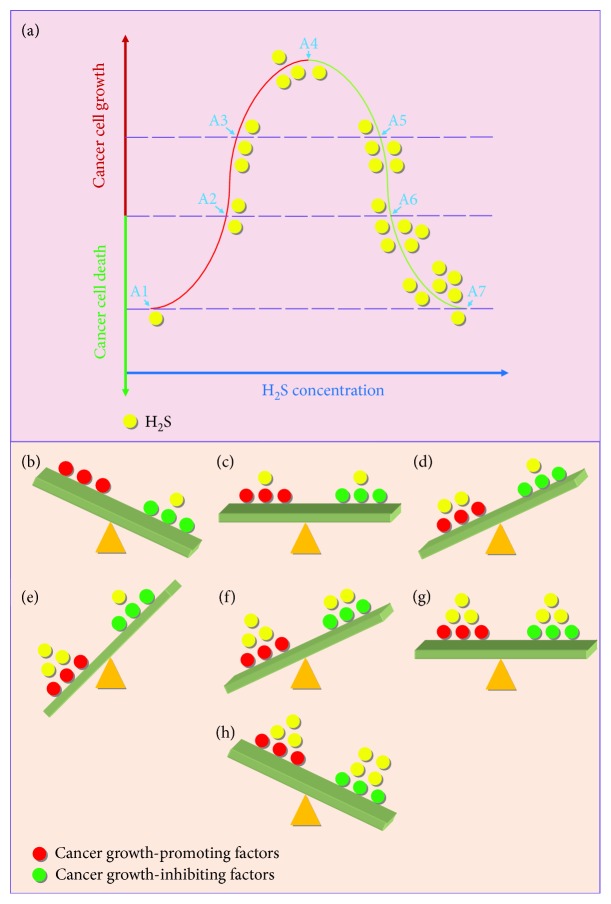
A proposed mechanism of the effect of H_2_S on the growth and death of cancer. (a) A normal distribution curve is employed to explain the effect of H_2_S on the development of cancer. Endogenous H_2_S or relatively low levels of exogenous H_2_S could promote cancer cell growth, while knockdown/knockout of the expression of H_2_S-generating enzyme or exposure of relatively high concentrations of H_2_S could induce cancer cell death. (b, A1) Downregulation of endogenous H_2_S induces cancer cell death. (c, A2) A certain concentration of H_2_S induces growth arrest in cancer cells. (d, A3) Normal level of H_2_S in cancer cells promotes cancer cell growth. (e, A4) Treatment with relatively low levels of exogenous H_2_S could exert optimal effects on the growth of cancer cells. (f-h, A5-A7) Along with the increase in the levels of exogenous H_2_S, the growth of cancer cells is gradually decreased. It is worth noting that the procession of cancer cells is theoretically the same between A1 and A7, A2 and A6, and A3 and A5.

Recent studies indicate that treatment with exogenous H_2_S could protect against a number of liver diseases, including hepatic fibrosis, liver cirrhosis, NAFLD/NASH, and hepatotoxicity. Novel H_2_S releasing/stimulating reagents can be designed and applied to enhance the therapeutic effects. An increasing number of evidence suggests that endogenous H_2_S or relatively low levels of exogenous H_2_S can promote the growth of HCC cells, while treatment with higher concentrations of H_2_S for a relatively long period may exhibit anticancer effects. We speculate that there is a delicate balance between the pro- and anticancer effects induced by H_2_S (Figure 2). Therefore, inhibition of the generation of endogenous H_2_S or administration of relatively high level of exogenous H_2_S could be effective in suppressing tumor growth. In addition, H_2_S could attenuate hepatic I/R injury in several ways, such as antioxidation, anti-inflammation, antiapoptosis, and AKT activation. Nevertheless, another study has shown that the increases in endogenous H_2_S exacerbate hepatic I/R injury, suggesting that increased levels of H_2_S may exhibit opposite effects. Furthermore, inhibition of CSE could alleviate ALF through upregulation of antioxidant and antiapoptotic defense in the liver. Novel inhibitors that target H_2_S-generating enzymes could be designed and applied in the treatment of ALF.

In conclusion, with a deeper understanding of the precise mechanisms behind the roles of H_2_S in liver health and disease, H_2_S could be a promising therapeutic target for further preclinical and clinical research.

## Figures and Tables

**Table 1:** Protective effects of H_2_S on hepatic fibrosis.

Experimental models	Effects	Proposed mechanisms	Refs.
Hepatic fibrosis *in vivo* (rat)	NaHS (56 *μ*mol/kg/day) attenuates CCl_4_-induced hepatic fibrosis	Reduction of liver expression levels of AGTR1	[20]
Hepatic fibrosis *in vivo* (rat)	NaHS solution (10 mmol/kg body weight) shows protective effects on CCl_4_-induced hepatic fibrosis	Decreased expression of p38 and increased expression of phospho-Akt	[88]
Hepatic fibrosis *in vivo* (rat)	NaHS solution (10 mmol/kg body weight) attenuates CCl_4_-induced hepatic fibrosis and ECM expression	Induction of cell cycle arrest and apoptosis in activated hepatic stellate cells	[89]
Hepatic fibrosis *in vivo* (rat)	NaHS (56 *μ*mol/kg/day) attenuates CCl_4_-induced hepatic fibrosis	Reduction of the expression of TGF-*β*1 and sediment of ECM in the liver tissues	[90]
Hepatic fibrosis *in vitro* (rat)	DATS (an H_2_S donor, 10 *μ*M) reduces H_2_O_2_-induced upexpression of fibrotic protein in HSCs	Unknown	[91]

CCl_4_: carbon tetrachloride; AGTR1: angiotensin II type 1 receptor; TGF-*β*1: transforming growth factor-*β*1; H_2_O_2_: hydrogen peroxide.

**Table 2:** Protective effects of H_2_S on hepatic I/R injury.

Experimental models	Effects	Proposed mechanisms	Refs.
Hepatic I/R *in vivo* (rat)	NaHS (14 *μ*M/kg, 30 min prior to I) attenuates the severity of liver injury and inhibits the production of lipid peroxidation, serum inflammatory factors, and apoptosis-related proteins	Antioxidant and antiapoptotic activities	[21]
Hepatic I/R *in vivo* (mouse)	H_2_S (100 ppm, 5 min prior to R) protects the liver against I/R injury	Reduction of apoptosis, necrosis, and inflammation	[119]
Hepatic I/R *in vivo* (rat)	GYY4137 (an H_2_S donor, 133 *μ*M/kg, 1 h prior to I) attenuates the reduced cell viability and the increased apoptosis induced by hepatic I/R	Activation of the Akt pathway regulated by miR-21	[120]
Hepatic I/R *in vivo* (rat)	NaHS (12.5, 25, and 50 *μ*M/kg, 5 min prior to I) reduces liver damage after perioperative I/R injury	Inhibition of MPTP opening and the activation of Akt-GSK-3*β* signaling	[121]
Hepatic I/R *in vivo* (rat)	NaHS (20 *μ*M/kg, 30 min prior to I) reduces hepatic I/R injury in the young rats	Activation of the Nrf2 signaling pathway	[122]
Hepatic I/R *in vivo* (rat)	NaHS (5 mg/kg/d for 11 days) protects against cognitive impairment in rats undergoing hepatic I/R	Reduction of neuroinflammation in the hippocampus	[123]
Hepatic I/R *in vivo* (mouse)	NaHS (1 mg/kg prior to R) ameliorates hepatic I/R injury by direct and indirect antioxidant activities and by accelerating hepatic regeneration	Via mechanisms involving Nrf2 and Akt-p70S6k	[124]
Hepatic I/R *in vivo* (rat)	NaHS (5 mg/kg/d for 11 days) exerts a protective effect on hepatic I/R-induced cognitive impairment	May be associated with the NR2B subunit of the NMDA receptors	[125]
Hepatic I/R in vivo (mouse)	NaHS (1.5 mg/kg, 1 h prior to I) protects against hepatic I/R injury	Partly through AKT1 activation	[126]
Hepatic I/R in vivo (mouse)	NaHS (14 and 28 *μ*M/kg, 30 min prior to I) attenuates hepatic I/R injury	Partly through regulation of apoptosis via inhibiting JNK1 signaling	[127]
Hepatic I/R in vivo (rat)	NaHS (28 *μ*M/kg, prior to R) attenuates hepatic I/R-induced renal and cardiac injury	Reduction of myocardial and renal inflammation and oxidative potential	[128]
Hepatic I/R *in vivo* (mouse)	Na_2_S (an H_2_S donor, 1 mg/kg, 5 min prior to R) protects the murine liver against I/R injury	Upregulation of intracellular antioxidant and antiapoptotic signaling pathways	[30]

MPTP: mitochondrial permeability transition pore; GSK-3*β*: glycogen synthase kinase-3 beta; Nrf2: nuclear factor erythroid 2-related factor 2; NMDA: NR2B subunit of N-methyl-D-aspartate; JNK1: c-Jun N-terminal kinase 1.

**Table 3:** Protective effects of H_2_S on NAFLD/NASH.

Experimental models	Effects	Proposed mechanisms	Refs.
NAFLD *in vivo* (mouse)	NaHS (56 *μ*mol/kg/day) attenuates HFD-induced NAFLD	Activation of liver autophagy via the AMPK-mTOR pathway	[138]
NAFLD *in vivo* (mouse)	NaHS (50 *μ*mol/kg/day) mitigates HFD-induced NAFLD	Improvement of lipid metabolism and antioxidant potential	[49]
NAFLD *in vivo* (mouse)	NaHS (14 *μ*mol/kg) attenuates concanavalin A-induced hepatitis	Inhibition of apoptosis and autophagy partly through activation of the PI3K-AKT1 signaling pathway	[139]
NASH *in vivo* (rat)	NaHS (28 *μ*mol/kg/day) attenuates MCD-induced NASH	Possibly through abating oxidative stress and suppressing inflammation	[22]
NAFLD *in vivo* (mouse)	SPRC (an H_2_S donor, 40 mg/kg/day) exerts a novel protective effect on MCD-induced NAFLD	Antioxidative effect through the PI3K/Akt/Nrf2/HO-1 signaling pathway	[50]

AMPK: adenosine monophosphate-activated protein kinase; mTOR: mammalian target of rapamycin; PI3K: phosphatidylinositol 3-kinase; MCD: methionine-choline-deficient; HO-1: heme oxygenase-1.

**Table 4:** Protective effects of natural sulfur-containing agents on hepatotoxicity.

Experimental models	Effects	Proposed mechanisms	Refs.
Hepatotoxicity *in vivo* (rat)	DATS (40 and 80 mg/kg, orally) protects against valproate-induced hepatotoxicity	Antioxidative, anti-inflammatory, and antiapoptotic properties	[159]
Hepatotoxicity *in vivo* (rat)	DADS (10 ml/kg/day) attenuates acetaminophen-induced acute hepatotoxicity	Possibly via the reduction of oxidative stress-mediated JNK activation and the suppression of inflammatory responses	[160]
Hepatotoxicity *in vivo* (mouse)	AMDS (50 mg/kg/day) protects against acetaminophen-induced hepatotoxicity	Through the strong attenuation of the CD45 expression and HNE formation	[161]
Hepatotoxicity *in vivo* (rat)	DATS (80 mg/kg/day) ameliorates arsenic-induced hepatotoxicity	Abrogation of oxidative stress, inflammation, and apoptosis	[162]
Hepatotoxicity *in vivo* (rat)	DADS (2 ml/kg/day) protects against carbon tetrachloride-induced hepatotoxicity	Through activation of Nrf2	[163]
Hepatotoxicity *in vivo* (rat)	DAS (200 mg/kg/day) ameliorates ferric nitrilotriacetate-induced hepatotoxicity	Unknown	[164]
Hepatotoxicity *in vivo* (mouse)	DATS (40 mg/kg) protects against isoniazid and rifampin-induced hepatotoxicity	Reduction of oxidative stress and activation of Kupffer cells	[165]

AMDS: allyl methyl disulfide; HNE: human neutrophil elastase.
